# Whole genome-wide association study reveals genetic insights into leaf spot disease resistances and seed germination/dormancy in peanut

**DOI:** 10.3389/fpls.2026.1838203

**Published:** 2026-06-10

**Authors:** Jie Zhang, Kelly Chamberlin, Ming Li Wang, Josh Clevenger, Phat Dang, Ye Chu, Corley Holbrook, Peggy Ozias-Akins, Charles Y. Chen

**Affiliations:** 1 Department of Crop, Soil, and Environmental Sciences, Auburn University, Auburn, AL, United States; 2 USDA-ARS Peanut and Small Grains Research Unit, Stillwater, OK, United States; 3 USDA-ARS Plant Genetic Resources Conservation, Griffin, GA, United States; 4 HudsonAlpha Institute for Biotechnology, Huntsville, AL, United States; 5 USDA-ARS National Peanut Research Laboratory, Dawson, GA, United States; 6 Department of Horticulture, University of Georgia, Tifton, GA, United States; 7 USDA-ARS Crop Genetics and Breeding Research Unit, USDA-ARS, Tifton, GA, United States

**Keywords:** *Arachis hypogaea* L., genome-wide association study (GWAS), leaf spot resistance, seed dormancy, seed germination

## Abstract

Peanut (*Arachis hypogaea* L.) is an important crop in the world, serving as a key source of edible oil and protein. Comprehensive genomic and phenotypic analyses were conducted on 87 accessions from the U.S. peanut mini-core collection using 217 Gb of high-quality resequencing data to identify the candidate genes and markers that underlie the leaf spot resistance and seed dormancy in peanuts. A total of 87,726 SNPs were identified and mapped across 20 chromosomes, revealing a higher SNP density in the B subgenome (35.55 SNPs/Mb) compared to the A subgenome (33.26 SNPs/Mb). Phylogenetic, population structure, and principal component analyses consistently partitioned the accessions into three distinct gene pools designated as Group 1, 2, and 3. Group 1, comprising primarily *Arachis hypogaea*, included 28 genotypes; Group 2, mainly *fastigiata* types, comprised 18 accessions; while Group 3, displaying the highest diversity, contained mixed genotypes from the other groups. Linkage disequilibrium analysis indicated an LD decay distance of approximately 63.1 kb, confirming that the marker density was sufficient for GWAS. Significant SNP associations at a suggestive threshold of p< 1.14 × 10^−1^ were identified for leaf spot, seed germination and dormancy agronomic traits. As a result, three candidate genes were identified: *Ah11g381400*, homologous to *Arabidopsis* ATE1, was associated with early leaf spot resistance; *Ah16g445600*, a homolog of ERF34, was linked to late leaf spot resistance; and *Ah19g214100*, homologous to ICE1, emerged as a central regulator affecting both germination and dormancy. These findings provide actionable targets for marker-assisted selection to enhance disease resilience and seed quality in breeding programs.

## Introduction

Peanut is an important oil and protein crop that originated in South America. Although the genus *Arachis* contains about 80 species, only one species, *Arachis hypogaea* has been domesticated as cultivated peanut. It is now widely grown, particularly in China, India, African countries, and the United States (Zhao et al., 2011). The cultivated peanut is an allotetraploid with 20 chromosome pairs (2n = 4x = 40), arising from the genome doubling of an interspecific hybrid within the section *Arachis*, which resulted from the crossbreeding of two older diploid ancestor species *Arachis duranensis* and *Arachis ipaensis* genomes (Chen et al., 2019). For its high nutritional value, *Arachis hypogaea* is extensively utilized in food products, oils, and animal feed (Zhao et al., 2011). Studies have shown that peanut kernels contain approximately 25% crude protein and 46% lipid content, making them a rich source of protein and oil for human consumption and industrial applications (Ingale and Shrivastava, 2011). Additionally, peanut meal, a by-product of oil extraction, is widely used as animal feed due to its high protein content (Zhao et al., 2023).

The economic importance *of Arachis hypogaea* has driven extensive research efforts to understand its origin, domestication and genetic architecture, particularly in yield, quality and resistance to biotic and abiotic stresses. Click or tap here to enter text. Large-scale genomic resources, including variation mapping, evolutionary analyses, and pangenome studies, have substantially improved the resolution of trait dissection and accelerated locus discovery for agronomic traits related to yield, quality, and stress adaptation (Lu et al., 2024; Guo et al., 2024; Zheng et al., 2024; Zhao et al., 2025). Among these traits, resistance to leaf spot and seed dormancy are of critical importance for sustainable peanut production and quality improvement (Clevenger et al., 2018; Han et al., 2018; Liang et al., 2018; Chu et al., 2019; Kumar et al., 2020; Chavarro et al., 2020; Wang et al., 2022). Understanding the genetic basis of these complex traits is key to improving peanut productivity and resistance of new cultivars.

Leaf spot, caused by fungal pathogens *Cercospora arachidicola* (early leaf spot) and *Cercosporidium personatum* (late leaf spot), is one of the most devastating diseases in peanut cultivation (Dang et al., 2021), leading to significant yield losses and increased reliance on chemical fungicides. Extensive research has been studied in this field. Earlier studies developed a comprehensive genetic linkage map comprising 248 marker loci, leading to the identification of 48 quantitative trait loci (QTLs) associated with disease resistance. These included 6 QTLs for tomato spotted wilt virus (TSWV), 22 for early leaf spot (ELS), and 20 for late leaf spot (LLS) resistance (Khera et al., 2016). More recently, three consistent ELS resistance QTLs (*qELS.A03_1.1, qELS.A03_1.2, and qELS.B03*) were identified, with *qELS.A03_1.1* corresponds to a previously reported LLS resistance region in GPBD4 (Chu et al., 2019). Additionally, major QTLs associated with resistance to LLS have been mapped on chromosome A03, including a common quantitative trait locus (QTL) shared with rust disease (LLS_QTL1_/Rust_QTL_) (Ahmad et al., 2020).

Seed dormancy is an important trait in crop domestication, playing a key role in preventing pre-harvest sprouting (PHS) and minimizing yield losses in various crops (Bewley and Black, 1985; Shelar et al., 2014; Nonogaki et al., 2018). Research has demonstrated significant variability in seed dormancy among different peanut accessions and botanical varieties. The botanical variety *hypogaea* exhibited stronger dormancy compared to other botanical varieties (Wang et al., 2012; Silva et al., 2017). Further study showed that seed dormancy in peanut has been linked to two major QTLs on chromosomes A04 and A05, with a candidate gene identified (*Arahy.KB746A*, ethylene-responsive transcription factor), providing opportunities for marker-assisted breeding to improve dormancy and reduce preharvest sprouting (Wang et al., 2022). Our previous study identified a significant QTL on chromosome A05, explaining 20% of the phenotypic variation in seed dormancy (Patel et al., 2022). A recent study using multi-locus and single-locus GWAS analysis with the groundnut mini-core collection and 58K SNP array data identified 47 significant SNP-trait associations (9 from ML-GWAS and 38 from SL-GWAS), including a validated QTL on chromosome B02 (*qFSD-B02-1*) and 9 key candidate genes regulating ABA/GA pathways for fresh seed dormancy improvement (Bomireddy et al., 2024).

Genome-wide association study (GWAS) is a powerful tool that employs linkage disequilibrium (LD) to identify genetic variants associated with phenotypic traits in large populations, enabling the detection of loci contributing to specific traits (Mackay and Powell, 2007). In peanut, most previous studies have focused on mapping quantitative trait loci associated with yield-related parameters, including seed weight, seed size, pod length, pod weight, and other productivity-related traits (Shirasawa et al., 2012; Chen et al., 2016; Zhang et al., 2019). Building on this foundation, recent high-impact studies have substantially advanced peanut GWAS resources. A large-scale genomic variation map constructed from 390 accessions identified 22,309 significant associations across 28 agronomic traits, including loci governing plant architecture and oil biosynthesis (Lu et al., 2024). Combining chloroplast and whole-genome sequencing with linkage mapping and GWAS revealed candidate genes and genomic regions associated with flowering pattern, pod and seed weight, and oil content, while clarifying evolutionary diversification within cultivated peanut (Zheng et al., 2024). Alongside these comprehensive genomic analyses, targeted studies continue to find important regions for key traits. For instance, recent work has successfully identified five loci on chromosome B06 influencing yield components, and two loci on chromosome A08 related to protein and oil content (Guo et al., 2024).

In our previous leaf spot resistance study, 46 QTLs were identified with phenotypic variation explained (PVE) from 10.19 to 24.11%, including 18 QTLs for resistance to ELS and 28 QTLs for LLS (Zhang et al., 2020). Additionally, using the same genetic diversity panel, we also investigated seed germination and dormancy traits and revealed genomic regions associated with these critical seed characteristics (Patel et al., 2022). However, these previous studies were conducted using SNP array-based genotyping platforms, which provided limited genomic resolution for precise mapping of trait-associated regions and more accurate identification of candidate genes.

To further reveal the genetic architecture in yield, quality and resistance to biotic and abiotic stresses, we performed whole genome association study for 2 disease-related traits, and 4 seed germination and dormancy related traits in 87 peanut accessions from the U.S. mini-core collection and identified 87,726 SNPs. Our objectives were to: (1) investigate the population structure and genetic diversity, (2) identify genomic regions associated with multiple important traits, including early and late leaf spot resistance and seed dormancy related traits through GWAS analysis, and (3) determine candidate genes within significant loci and analyze their haplotype variations to provide insights into the genetic architecture of these agronomically important traits.

## Methods

### Phenotype evaluation

In this study, 87 accessions of the US mini-core collection were phenotyped. These genotypes were comprised of three botanical varieties: *hypogaea, fastigiata, vulgaris* and *peruviana* (**Supplementary Table 1**) (Holbrook and Dong, 2005). The geographic origins of these accessions are diverse, such as the United States, China, India, Nigeria, Argentina, Brazil, South Africa, Sudan, and Zambia, which are the main peanut-growing areas worldwide.

The evaluation procedures for Early Leaf Spot (ELS) and Late Leaf Spot (LLS) phenotypes were conducted as described in our previous study (Zhang et al., 2020). ELS assessments were carried out in 2013 and 2014 at the E.V. Smith Research Center of Auburn University, located in Shorter, AL (32°29′N, 85°53′W). LLS evaluations were conducted in 2013, 2014, and 2015 at the Wiregrass Research and Extension Center of Auburn University in Headland, AL (31°22′N, 85°19′W). For both diseases, severity was visually assessed on a plot basis using the Florida scale, which ranges from 1 (no disease) to 10 (plant death) (Chiteka et al., 1988).

The procedures for evaluating seed dormancy, germination, and grading followed previously published protocols (Patel et al., 2022). In 2010, peanut seeds were planted in Dawson, GA, USA, using a randomized complete block design with three replications in two-row, 10-foot plots. Irrigation ensured optimal soil moisture, and crop management adhered to standard practices, including nutrient application (N:P:K = 120:26:33 kg ha−1) and pest control. A small peanut was used for harvesting, and pods were dried to 10% moisture for further evaluation. For germination testing, fungicide-treated seeds were evaluated using duplicate sets of 25 seeds on moistened germination paper in controlled growth chambers (30 °C for 12 h with 8 h light, 20 °C for 12 h dark, 85% relative humidity). Germination was recorded at 7, 14, and 21 days, with non-germinated and decayed seeds classified as non-viable.

### DNA isolation, genome resequencing and SNP detection

For each peanut accession, we collected young leaf tissue for DNA extraction using the CTAB method, and we constructed paired-end whole genome sequencing libraries with insert sizes ranging from 300 bp to 500 bp. The libraries were sequenced using the Illumina NovaSeq 6000 platform, generating a total of 217 Gb of high-quality sequences, with approximately 2.5 Gb per sample. *The Arachis hypogaea* reference genome was utilized for mapping (Bertioli et al., 2019), and genomic variations were identified using the Khufu platform from HudsonAlpha institute.

### Phylogenetic tree and population structure

To clarify the phylogenetic relationship of mini-core accessions, we used VCF2Dis (https://github.com/BGI-shenzhen/VCF2Dis) to calculate P-distance matrix. Subsequently, FastMe2.0 (Lefort et al., 2015) used to infer the phylogenetic relationship between individuals using the Neighbor-Joining (NJ) method. Finally, the phylogenetic tree was visualized by iTOL (https://itol.embl.de/). Population structure was calculated with Admixture, and the number of assumed genetic clusters (K) ranged from 1 to 10, with 10,000 iterations performed for each run. Additionally, we conducted the Principal Component Analysis (PCA) to assess the genetic structure using GCTA 1.24.2 (http://cnsgenomics.com/software/gcta/pca.html) (Yang et al., 2011).

Based on the results of the population genetic structure analysis, we conducted ANOVA to assess the phenotypic differences among the three groups. Subsequently, multiple comparisons were performed using the Honestly Significant Difference (HSD) method.

### Population genetics analysis

We performed population genetics analyses to measure genetic differentiation and variation across the genome, fixation statistics (*F_ST_*) to assess population differentiation and nucleotide diversity (*θ_π_*) to quantify genetic variation within populations. Both were calculated in VCFtools v0.1.17 (Danecek et al., 2011), with sliding windows of 100 Kb and step size of 20 Kb.

### Linkage-disequilibrium analysis

To analyze the decay of linkage disequilibrium (LD), we used PLINK software (Purcell et al., 2007) to calculate the LD coefficient (r^2^) between pairwise high-quality SNPs. The following parameters were applied: --ld-window-r2 0, --ld-window 99999, and --ld-window-kb 1000. The calculated r^2^ values were then used to estimate LD decay by plotting r^2^ against physical distance and determining the point at which LD decreased to a baseline level.

### GWAS analysis

Prior to GWAS, SNPs were quality-filtered, and only variants with minor allele frequency (MAF) ≥ 0.05 and missing rate ≤ 0.2 were retained. The final filtered dataset comprised 87,726 SNPs and was used for association analyses of leaf spot disease and seed germination and dormancy traits. To account for accession imbalance due to geographical distribution, we applied the genome-wide efficient mixed-model association (GEMMA v0.98.3) software package (Zhou and Stephens, 2012). The mixed linear model (MLM) analysis was performed using the following equation:


y= Xα+ Sβ+ Kμ+ e

Where y is the vector of phenotype, *α* and *β* are fixed effects representing marker and non-marker effects respectively; *μ* is random effects. *X*, *S* and *K* are the incidence matrices corresponding to *α*, *β* and *μ*, respectively, and *e* is the vector of random residual effects. To correct for population structure, the top three principal components (PCs) were included in the S matrix, and a simple matching coefficient matrix was used to construct the K matrix. All analyses were conducted using GEMMA. A genome-wide significance threshold was determined using a uniform threshold of 1/n, where n was the effective number of genomic variations (calculated as 87,726), following previous peanut GWAS that applied the same empirical threshold (Li et al., 2022; Xu et al., 2025). Marker effects (*β*) were tested for significance using the Wald test in GEMMA. From the Manhattan plots, all SNPs passing the significance threshold were considered significant and retained for downstream analyses. To evaluate the fit between observed and expected values of quantitative traits, Manhattan plot results were validated using Q-Q plots.

### Identification of the candidate genes in the GWAS associated loci

To detect the candidate genes, we defined the candidate region as spanning 1 megabase (1 Mb) upstream and downstream of each significant SNP. Annotated genes and SNPs within these regions were extracted and analyzed. For each gene of candidate region, which was functionally annotated using TAIR genomic databases website (https://www.arabidopsis.org/), and their potential functions relevance to the trait of interest were retained.

### Haplotype analysis

For each SNP within the candidate region, haplotype-specific phenotypic associations were evaluated using a non-parametric framework. The Kruskal–Wallis test (significance set at p< 0.05) was used to assess overall differences in phenotype distributions among haplotype groups. When significant differences were detected, Dunn’s *post hoc* test was performed for pairwise comparisons.

## Results

### Population properties of mini-core peanut collection

To further reveal the genomic variation of mini-core peanut accessions, we generated a total of 217 Gb high-quality resequencing data from 87 accessions collected worldwide (**Supplementary Table 1**). We subsequently identified a total of 87,726 SNPs, and they widely distributed on 20 chromosomes. To investigate the SNP distribution between the A and B subgenomes, we calculated SNP densities, finding that the A subgenome exhibited 33.26 SNPs/Mb, while the B subgenome had 35.55 SNPs/Mb. This indicates that the B subgenome has a higher SNP density compared to the A subgenome, with chromosome 19 showing particularly high SNP density (**Supplementary Figure 1**). Furthermore, the nucleotide diversity (*θ_π_*) within the two subgenomes was calculated, revealing that the nucleotide diversity of B-subgenome (1.54 × 10^−5^) is higher than A-subgenome (1.43 × 10^−5^).

**Figure 1:**
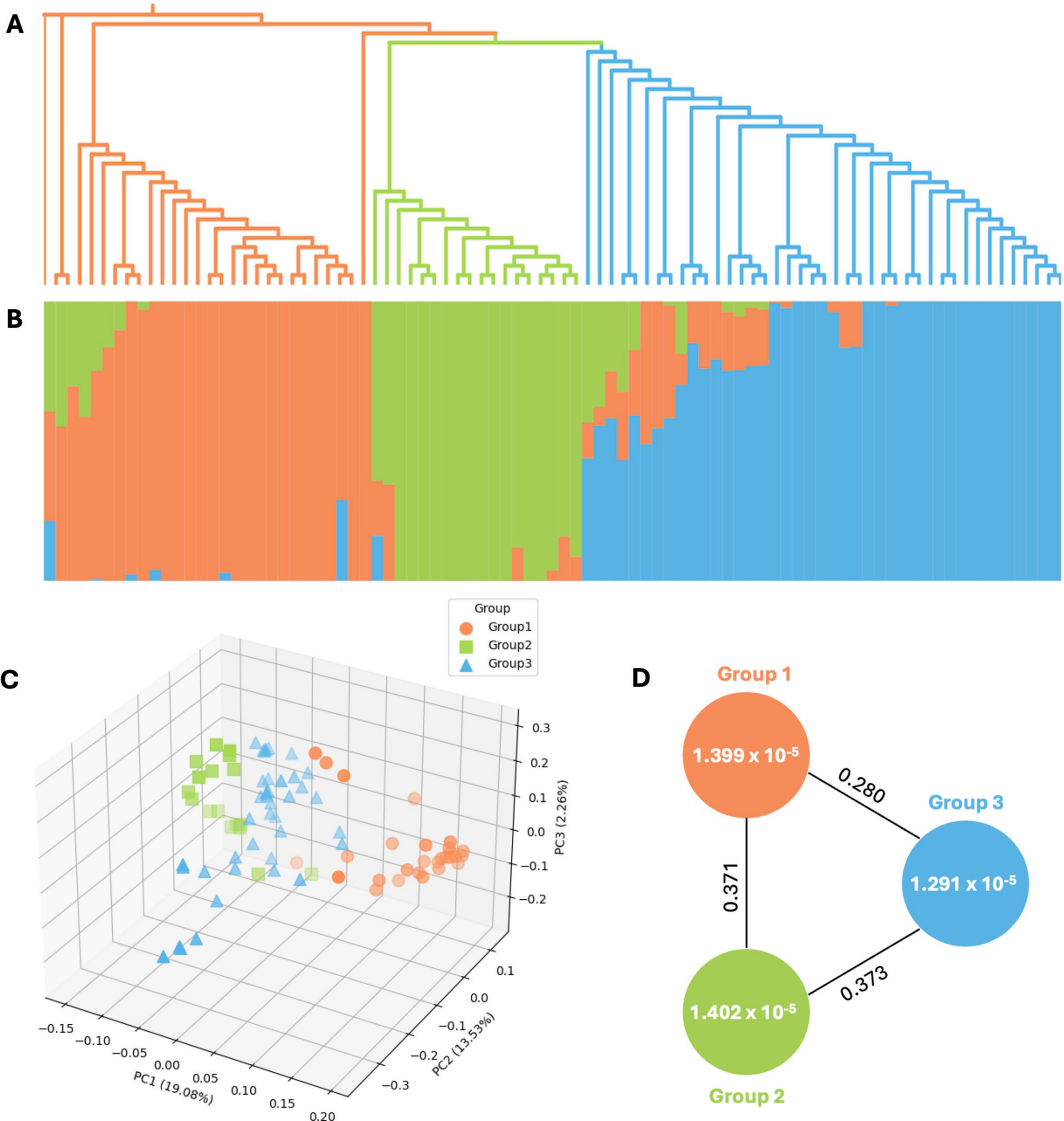
Phylogenetic tree, genetic structure, PCA. **(A)** Phylogenetic tree of the population, generated with 87,726 high-quality SNPs. **(B)** Structure analysis with K = 3. The y-axis quantifies cluster membership, and the x-axis represents different accessions. The orders and positions of accessions on the x axis are consistent with those in the phylogenetic tree. **(C)** PCA plot of the first three components (PC1, PC2 and PC3). **(D)** Genetic diversity and population differentiation across the three groups. The values in the circles represent the genetic diversity (*Θπ*) of the groups, and the values between the groups indicate population differentiation (*F*_ST_).

In order to uncover the phylogenetic relationships of mini-core collection, we performed phylogenetic tree construction, population structure, and PCA analysis (**Figure 1**). Three distinct groups were identified from the 87 mini-core accessions based on the NJ analysis designated here as G1, G2 and G3 (**Figure 1A**). These divisions were also supported by population structure analysis (**Figure 1B**) and PCA (**Figure 1C**). Group 1 is represented by *Arachis hypogaea* and contains 28 genotypes. Group 2 mainly consists of *fastigiata* types and includes 18 accessions. Group3, which has more diversity compared to the other two groups, spans a larger portion and includes other two group memberships, indicating a mixture of genotypes from both Group1 and Group 2.

The nucleotide diversity (*θ_π_*) values across the three subpopulations ranged from 1.291 to 1.402 × 10^-5^ (**Figure 1D**). Group 2 exhibited the highest nucleotide diversity, reaching 1.402 × 10^−1^, followed closely by Group 1 with 1.399 × 10^−1^. In contrast, Group 3 showed the lowest diversity at 1.291 × 10^−1^. These values indicate a comparable level of genetic variation within the groups. The fixation index values (*F_ST_*) among the three subpopulations ranged from 0.280 to 0.373, which revealed moderate to high levels of genetic differentiation. Specifically, the *F_ST_* between Group 1 and Group 2 was 0.371, between Group 2 and Group 3 was 0.373, and between Group 1 and Group 3 was 0.280. These results suggest that Groups 1 and 3 are relatively less differentiated compared to other pairwise comparisons, but overall, the populations exhibit substantial genetic structuring, which may reflect historical isolation or limited gene flow.

### Phenotypic analysis

**Figure 2:**
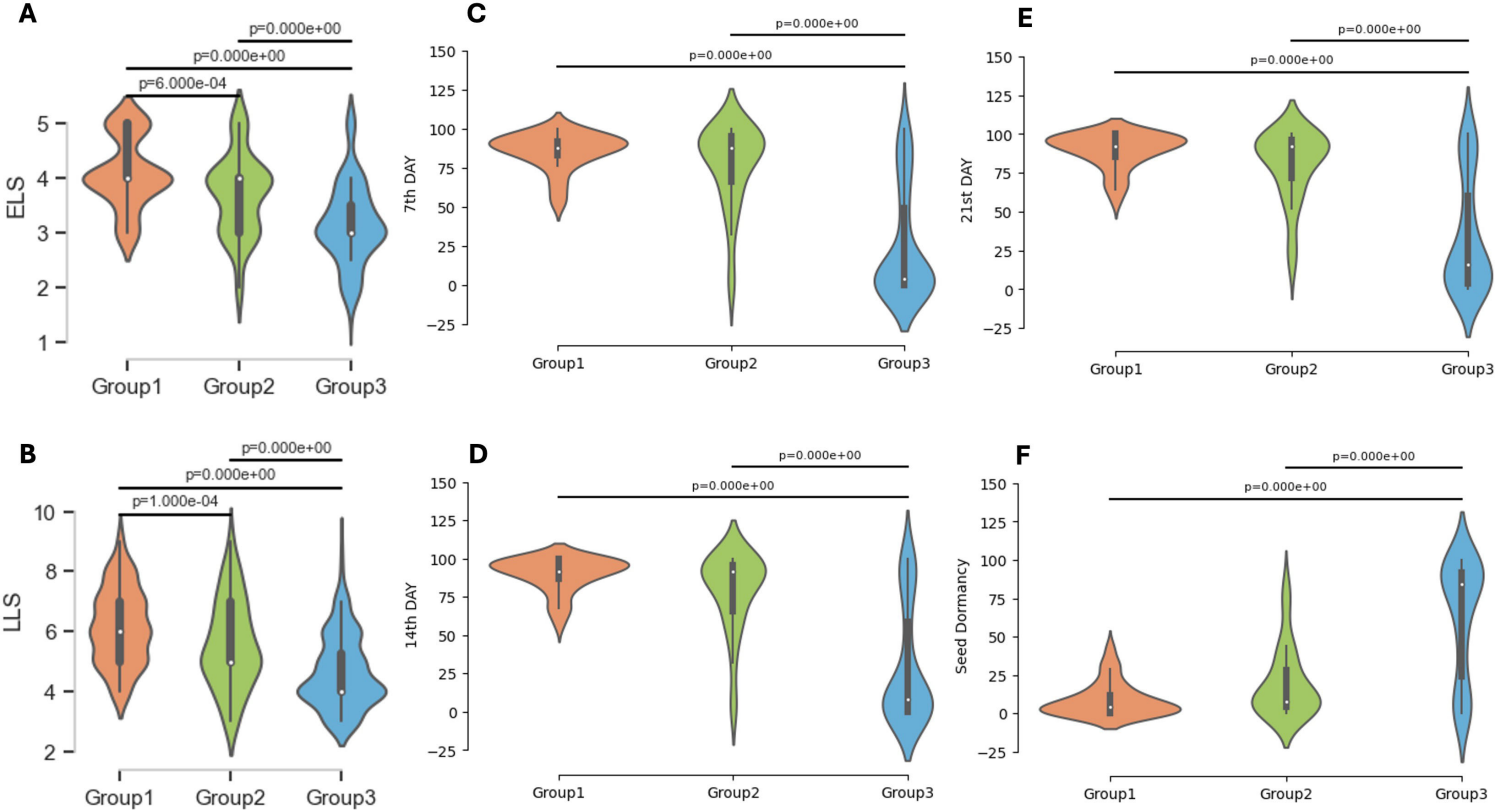
Phenotypic variation among three genetic groups for disease resistance and seed dormancy traits. **(A)** ELS resistance scores across three genetic groups (p<0.001). **(B)** LLS resistance scores among genetic groups showing significant differences (p<0.001). **(C)** Seed germination rates at 7 days post-planting across three genetic groups (p<0.001). **(D)** Seed germination rates at 14 days post-planting across three genetic groups (p<0.001). **(E)** Seed germination rates at 21 days post-planting across three genetic groups (p<0.001). **(F)** Overall seed dormancy levels among the three genetic groups (p<0.001).

To investigate how these genetic groups correspond to phenotypic variation, we performed ANOVA for key traits, followed by multiple comparisons using the HSD method. For disease resistance traits ELS and LLS, the results revealed significant differences (p< 0.001) for both traits. Tukey’s HSD test further confirmed these differences, highlighting clear phenotypic distinctions among the genetic subpopulations (**Supplementary Table 2** and **Figures 2A, B**). For seed dormancy-related traits, including seed dormancy and germination, significant differences were observed among the groups based on the number of germinated seeds recorded at 7, 14, and 21 days. Tukey’s HSD test showed that Group 3 consistently exhibited lower germination rates and higher dormancy compared to Groups 1 and 2 (**Supplementary Table 2**; **Figures 2C–F**).

### Analysis of linkage disequilibrium

The decay rate of LD was calculated as the pairwise correlation coefficient (*r^2^*) from the maximum value to the threshold value of 0.1. The LD decay distance was approximately 63.1 kb for all the accessions (**Supplementary Figure 2**). The availability of 87,726 SNPs exceeds the minimum requirement of 44,000 SNPs for GWAS, ensuring sufficient marker density for reliable association mapping.

### Genome wide association studies with quantitative traits and candidate genes identification and haplotype analysis

To dissect the genetic basis of agronomically important traits, we focused on the 6 traits, including 2 disease-related traits, and 4 seed germination and dormancy related traits. The multiple environmental traits phenotypic values (for multiple environmental traits) were used to perform GWAS with 87,726 SNPs. A threshold of p-value< 1.14 × 10^−1^ was employed to identify significant SNP markers associated with the target traits. In terms of disease resistance, we found 8 significant SNPs for early leaf spot (ELS) and 10 for late leaf spot (LLS). In the seed dormancy related traits, we identified 65 SNPs associated with 7-day germination, of which 22 were consistently validated in two independent phenotypic analyses. Likewise, 79 SNPs were associated with 14-day germination, with 29 validated across two separate evaluations. Similarly, for 21-day germination, we detected 70 SNPs, 32 of which were repeatedly confirmed in both phenotypic analyses. In addition, dormancy exhibited the highest number of significant SNPs (112), and notably, 40 of these SNPs were detected in all four dormancy-related traits (7-day, 14-day, 21-day, and dormancy). Among these 40, Chr10 and Chr20 contained the most associations, with 9 and 11 SNPs, respectively, underscoring their potential importance in controlling peanut seed dormancy (**Supplementary Table 3**). These findings also show that most of these SNPs are distributed in the B sub-genome. Our results provide insights into the genetic architecture of complex traits in peanut, highlighting both unique and overlapping loci associated seed dormancy, and disease resistance traits. These findings offer a valuable resource for further exploration of candidate genes and the development of marker-assisted selection strategies for peanut breeding programs.

### Leaf spot

**Figure 3:**
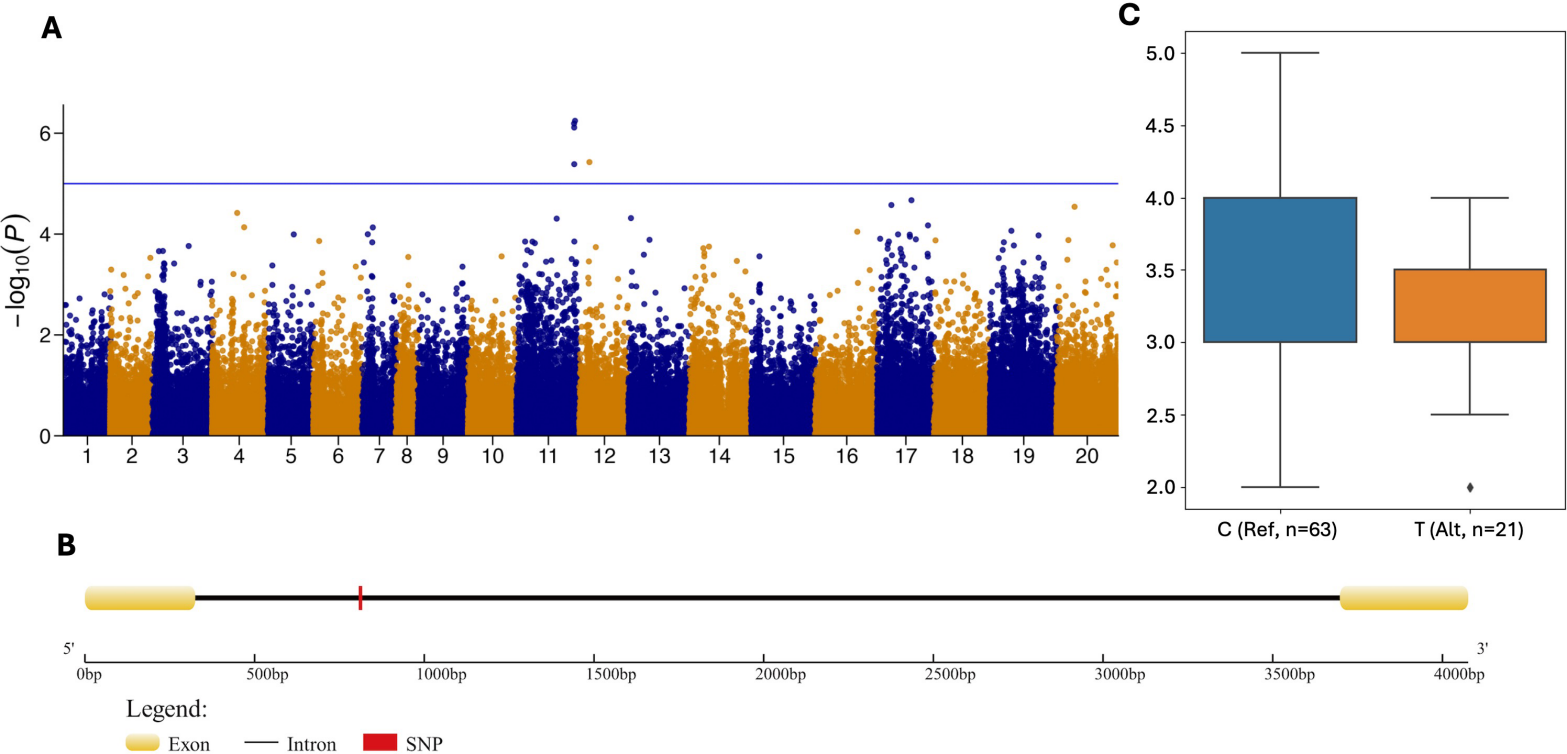
**(A)** Manhattan plot for ELS. The blue horizontal line indicates the suggestive significance threshold (-log_10_*P =* 4.94). **(B)** Gene structure of *Ah11g381400*, highlighting exons (yellow boxes), introns (black lines), and the significant SNP (red). **(C)** Haplotype analysis for ELS.

Four significant SNPs associated with ELS were identified on chromosome 11 (**Figure 3A**), and candidate regions were defined by extending 1 Mb upstream and downstream of each SNP. Within this candidate region, we identified *Ah11g381400*, which is homologous to the *Arabidopsis ATE1*(*AT5G05700*) gene, with the associated SNP located in the intronic region of the gene (**Figure 3B**). To further investigate the genetic basis underlying the ELS phenotype, we performed a haplotype analysis focusing on the SNP loci within the candidate region. Accessions were grouped based on the reference (C) or alternate (T) allele, resulting in 63 accessions with the C haplotype and 21 with the T haplotype. The average ELS value was 3.70 in the C group and 3.26 in the T group (**Figure 3C**), with a statistically significant difference (p = 7.526 × 10^−3^). Accessions with the T allele exhibited a 13.5% reduction in susceptibility relative to those with the C allele. Previous studies have shown that *Arabidopsis ATE1* mutants exhibit delayed leaf senescence and altered pathogen responses (Graciet et al., 2009; De Marchi et al., 2016), suggesting that *Ah11g381400* may represent an important candidate gene for further investigation into the ELS regulatory pathway, with the T allele conferring enhanced resistance.

**Figure 4:**
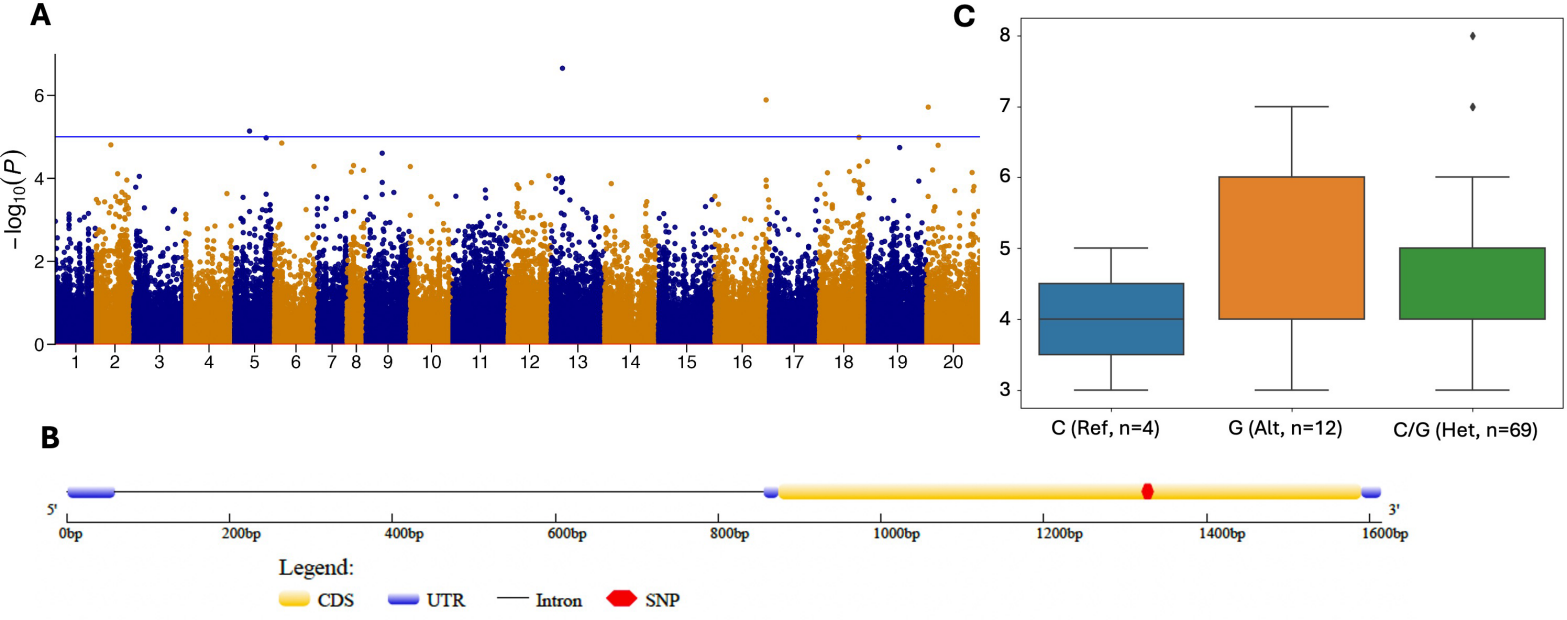
**(A)** Manhattan plot for LLS. The blue horizontal line indicates the suggestive significance threshold (-log_10_*P =* 4.94). **(B)** Gene structure of *Ah16g445600*, highlighting exons (yellow boxes), introns (black lines), and the significant SNP (red). **(C)** Haplotype analysis for LLS.

For the LLS phenotype, a significant SNP was identified on chromosome 16 (**Figure 4A**). Extending 1 Mb upstream and downstream of this SNP defined a candidate region that included the *Ah16g445600* gene, which is homologous to *Arabidopsis ERF34* (*AT2G44940*), a gene known to play critical roles in stress responses and senescence regulation (Park et al., 2022). The associated SNP was located in the coding sequence (CDS) region of *Ah16g445600* (**Figure 4B**). Among the analyzed accessions, the reference (C) haplotype was detected in four individuals, the alternative (G) haplotype in 12, and the heterozygous (C/G) genotype in 69. Haplotype-based phenotypic analysis revealed that individuals with the C haplotype exhibited an average LLS value of 4.0, compared with 5.3 for those with the G haplotype, while heterozygous (C/G) accessions displayed an intermediate value of 4.6 (**Figure 4C**). The disease index for the C haplotype was 32.5% lower than that for the G haplotype, indicating that the C allele confers enhanced resistance to LLS. These findings underscore the significant association of *Ah16g445600* with LLS and highlight its potential role, possibly involving *ERF34*-mediated pathways, in regulating leaf senescence and stress responses.

### Seed germination and dormancy

**Figure 5:**
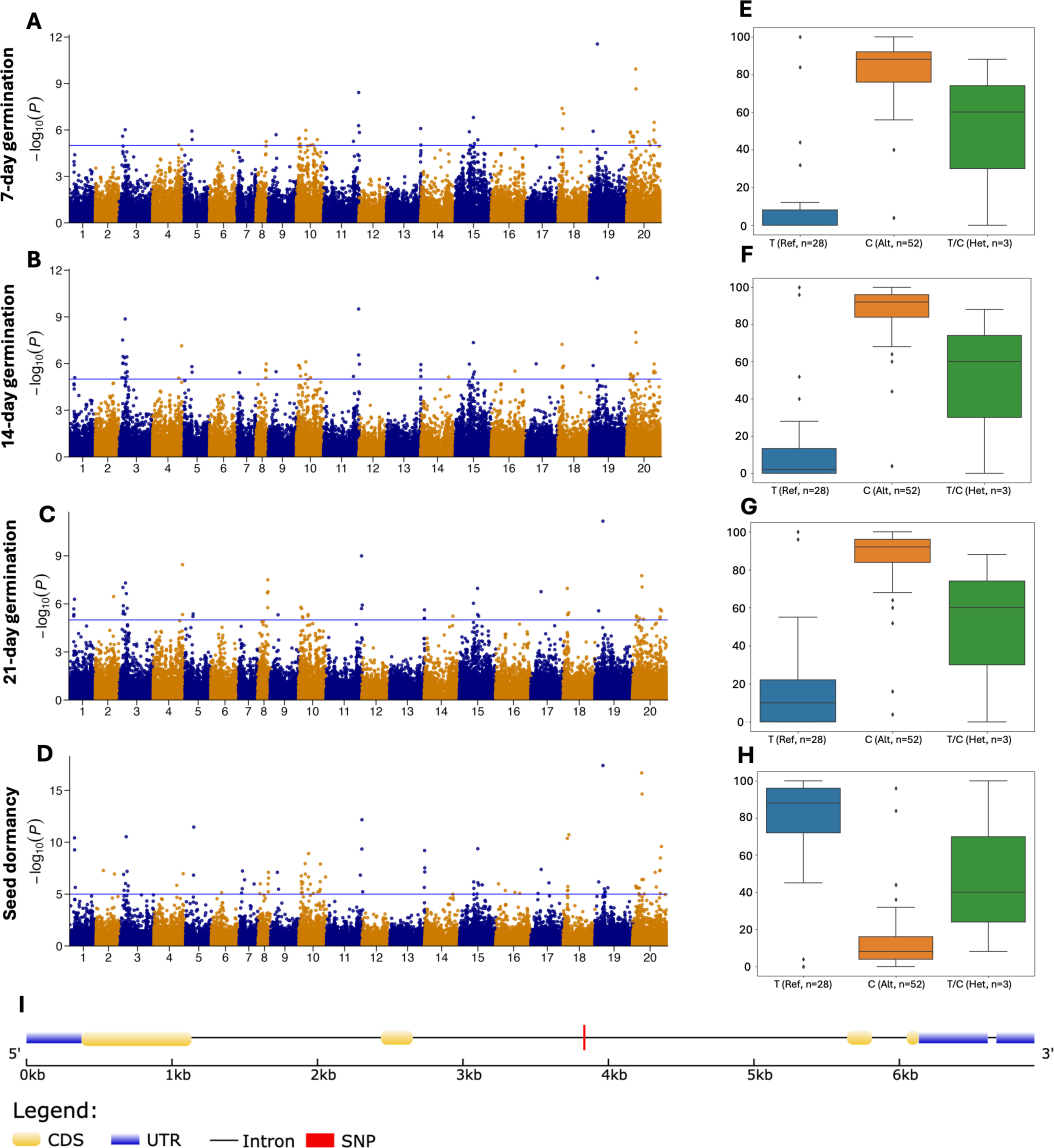
Genome-wide association study and haplotype analysis of seed germination and dormancy traits. **(A)** Manhattan plot of SNP associations with 7-day germination. **(B)** Manhattan plot of SNP associations with 14-day germination. **(C)** Manhattan plot of SNP associations with 21-day germination. **(D)** Manhattan plot of SNP associations with seed dormancy. **(E)** Box plot showing phenotypic differences among haplotypes for 7-day germination. **(F)** Box plot showing phenotypic differences among haplotypes for 14-day germination. **(G)** Box plot showing phenotypic differences among haplotypes for 21-day germination. **(H)** Box plot showing phenotypic differences among haplotypes for seed dormancy. **(I)** Gene structure of *Ah19g214100* (ICE1 homolog) showing the functional SNP position and genomic features (CDS, UTR, intron).

A total of 65, 79, and 70 significant SNPs were associated with germination at 7, 14, and 21 days, respectively, while 112 significant SNPs were detected for seed dormancy. Among these SNPs, an important candidate region common to all germination stages and dormancy traits contained the gene *Ah19g214100* (**Figures 5A–D**), a homolog of *Arabidopsis ICE1* (*AT3G26744*). The associated SNP was located in the intronic region of *Ah19g214100* (**Figures 5I**). This finding is consistent with recent studies demonstrating *ICE1*’s pivotal role in regulating ABA signaling and seed dormancy. For instance, Hu et al (Hu et al., 2019). reported that *ICE1* negatively modulates ABA-responsive genes to influence germination, while MacGregor et al (MacGregor et al., 2019). established *ICE1* as a key determinant of primary seed dormancy, with *ice1* mutants exhibiting enhanced dormancy phenotypes reversible by stratification. These functional parallels strongly implicate *Ah19g214100* as a central regulator of germination and dormancy.

Haplotype analysis of the *ICE1* homolog region revealed a consistent distribution pattern across all examined traits. The population comprised 52 individuals with the alternative (C) haplotype, 28 with the reference (T) haplotype, and 3 with the heterozygous (T/C) genotype. Statistical analysis using the Kruskal–Wallis test and Dunn’s *post hoc* test demonstrated significant phenotypic differences between the reference and alternative haplotypes across all traits (p< 0.05). Interestingly, we observed an inverse relationship between germination and dormancy phenotypes associated with these haplotypes (**Figures 5E–H**). For germination traits (7-day, 14-day, and 21-day), accessions carrying the alternative (C) haplotype exhibited significantly higher germination rates compared to those with the reference (T) haplotype. Conversely, in the dormancy assessment, reference (T) haplotype carriers showed higher dormancy levels, while alternative (C) haplotype carriers demonstrated reduced dormancy. This inverse relationship between germination efficiency and dormancy intensity mirrors the antagonistic roles of ABA and gibberellin signaling pathways in seed physiology. Overall, these results reinforce the notion that this locus may exert pleiotropic effects on both germination and dormancy, underscoring its potential significance for seed-related traits.

## Discussion

This study generated 217 Gb of high-quality resequencing data from 87 collected U.S. mini core peanut accessions and identified 87,726 SNPs distributed across 20 chromosomes. Additionally, we observed differential SNP distribution between subgenomes, with the B-subgenome exhibiting higher nucleotide diversity (1.54 × 10^−5^) compared to the A-subgenome (1.43 × 10^−5^). This aligns with previous studies suggesting that the B genome progenitor (*A. ipaensis*) possesses greater genetic diversity than the A genome progenitor (*A. duranensis*) (Bertioli et al., 2016; Chen et al., 2019).

The population structure analysis revealed three major genetic groups, representing different gene pools within cultivated peanut. Group 1, predominantly represented by *Arachis hypogaea*, and Group 2, mainly comprising *fastigiata* types, displayed distinct genetic backgrounds, while Group 3 appeared to be a mixture of these two pools.

The moderate to high *F_ST_* values (0.280 - 0.373) between these groups indicate substantial genetic differentiation, suggesting historically limited gene flow or potential adaptation to different environments or breeding objectives. Group 3 exhibited the lowest nucleotide diversity (1.291 × 10^−5^) compared to Groups 1 and 2, potentially indicating a narrower genetic base, which could be the result of more intensive selection during breeding or a founder effect. Importantly, our phenotypic analysis further validates these genetic groupings, with significant phenotypic differentiation among the three groups for multiple traits, particularly in disease resistance (ELS and LLS) and seed dormancy characteristics, with Group 3 exhibiting consistently higher dormancy and lower germination rates.

The GWAS analysis identified numerous significant SNPs associated with disease resistance, seed grade, and seed dormancy traits, providing insights into their genetic architecture. The identification of a higher number of significant SNPs in the B subgenome for seed dormancy-related traits suggests the predominant role of B subgenome in controlling these characteristics. This aligns with our observation of higher genetic diversity in this subgenome.

To identify candidate genes underlying significant GWAS signals, we adopted a strategy of examining regions extending 1 Mb upstream and downstream from each significant SNP (Yano et al., 2016; Fang et al., 2017). We acknowledge that this 2 Mb window is broad relative to the LD decay estimated in our dataset (~63.1 kb), and therefore may include non-causal genes. However, previous analysis of the U.S. peanut mini-core also reported long-range LD persistence (genome-wide half-decay distance ~3.78 Mb) (Otyama et al., 2019), indicating that LD extent can vary substantially depending on marker platform and population structure. Thus, we used the ±1 Mb window as a conservative discovery strategy to reduce false negatives. Future fine-mapping with larger populations and denser marker panels, combined with the integration of expression QTL (eQTL) data, would substantially narrow these intervals and facilitate the identification of causal variants with greater precision.

Our GWAS analysis identified key candidate genes for both disease resistance and seed dormancy traits. For ELS resistance, *Ah11g381400*, homologous to *Arabidopsis ATE1*, emerged as a promising candidate. *ATE1* regulates protein arginylation, affecting leaf senescence and pathogen responses (Yoshida et al., 2002; Graciet et al., 2010). In *Arabidopsis*, loss-of-function mutations in AtATE1 produce a delayed leaf senescence phenotype, with senescence progressing significantly more slowly than in wild-type plants under both age-dependent and dark-induced conditions (Yoshida et al., 2002). Beyond senescence, the N-end rule pathway mediated by ATE1 regulates diverse processes including seed germination, leaf and shoot development, and stress responses (Tasaki and Kwon, 2007). The connection between senescence regulation and disease resistance is mechanistically plausible: fungal pathogens such as *Cercospora arachidicola* actively exploit host senescence programs to facilitate colonization and sporulation, and plants with attenuated senescence frequently show enhanced resistance to necrotrophic and hemibiotrophic pathogens (Häffner et al., 2015). The associated SNP at *Ah11g381400* is intronic (**Figure 3B**), suggesting that the observed allelic effects may be mediated through regulatory mechanisms such as altered splicing or transcriptional modulation rather than direct amino acid changes. In our haplotype analysis, accessions carrying the T allele at the lead SNP exhibited a 13.5% reduction in ELS susceptibility relative to those carrying the C allele. These results suggest that this allelic variation may influence *Ah11g381400* expression or splicing, thereby modulating the rate of pathogen-accelerated senescence in inoculated leaves.

For LLS resistance, we identified *Ah16g445600*, homologous to *ERF34*, an AP2/ERF family transcription factor. It functions within ethylene signaling pathways that regulate both stress responses and senescence (Iqbal et al., 2017). *ERF34* belongs to a set of ERF transcription factors whose expression is altered under both age-dependent and dark-induced senescence, and all identified members of this group have been previously implicated in biotic and abiotic stress responses (Park et al., 2022). This functional background provides a mechanistic explanation for the LLS phenotype, because late leaf spot progression is accompanied by senescence-associated tissue damage. Accessions carrying the C haplotype at the candidate gene showed a 32.5% lower disease index compared to G haplotype carriers in our analysis. The lead SNP at this locus is located in the CDS region and is annotated as a synonymous variant (**Figure 4B**), with codon substitution from GTC to GTG (both encoding valine). Although it does not change amino acid identity, synonymous variants may still influence codon usage, translation efficiency, mRNA stability, or may be in linkage disequilibrium with nearby causal variants. Therefore, functional validation is still required to determine the causal mechanism.

For seed dormancy, *Ah19g214100*, homologous to *Arabidopsis ICE1*, was consistently identified across all germination timepoints. *AtICE1* is a *bHLH* transcription factor originally characterized for its role in cold stress signaling but subsequently identified as a key regulator of seed dormancy (Chinnusamy et al., 2003; MacGregor et al., 2019). In *Arabidopsis*, *ICE1* is expressed in the endosperm and contributes to determining primary dormancy depth, in part through negative regulation of ABA signaling during germination (MacGregor et al., 2019; Hu et al., 2019). In our population, the C haplotype was associated with higher germination rates and reduced dormancy compared with the T haplotype, consistent with ABA-gibberellin antagonism in seed physiology. The lead SNP at this locus is intronic (**Figure 5I**), suggesting a potential regulatory effect rather than a direct coding change. The pleiotropic association of this locus across all four dormancy-related traits (7-, 14-, and 21-day germination, and dormancy score) further supports *Ah19g214100* as a central candidate gene for dormancy regulation in peanut.

These findings provide valuable targets for marker-assisted selection to optimize both disease resistance and seed characteristics in peanut breeding programs. Overall, this study advances our understanding of genetic diversity and trait architecture in peanut, offering both fundamental insights into crop evolution and practical tools for crop improvement. The identified genetic candidate genes provide a foundation for developing peanut varieties with enhanced disease resistance and optimized seed characteristics to meet the challenges of sustainable agriculture. Further research will be essential to validate the specific genes responsible for these key agronomic traits.

## Data Availability

The genomic data associated with this study have been deposited in the NCBI database under BioProject accession number PRJNA1475457.

## Supplementary material

Supplementary Figure 1 Distribution of SNP markers in the 20 chromosomes. Horizontal axis represents physical distance along each chromosome. Vertical axis represents the 20 chromosomes of *Arachis hypogaea*; The color index represents the number of SNPs in 2.0 Mb window.

Supplementary Figure 2 Linkage disequilibrium (LD) decay over distance.

Supplementary Figure 3 Q–Q plot of observed vs. expected log10P values. **(A)** ELS. **(B)** LLS. **(C)** 7-day germination. **(D)** 14-day germination **(E)** 21-day germination **(F)** seed dormancy.
