# Supplementary figures and images for: Whole genome-wide association study reveals genetic insights into leaf spot disease resistances and seed germination/dormancy in peanut
Source: Front Plant Sci. 2026 Jun 10;17:1838203. doi: 10.3389/fpls.2026.1838203 (PMC13290452; doi:10.3389/fpls.2026.1838203)

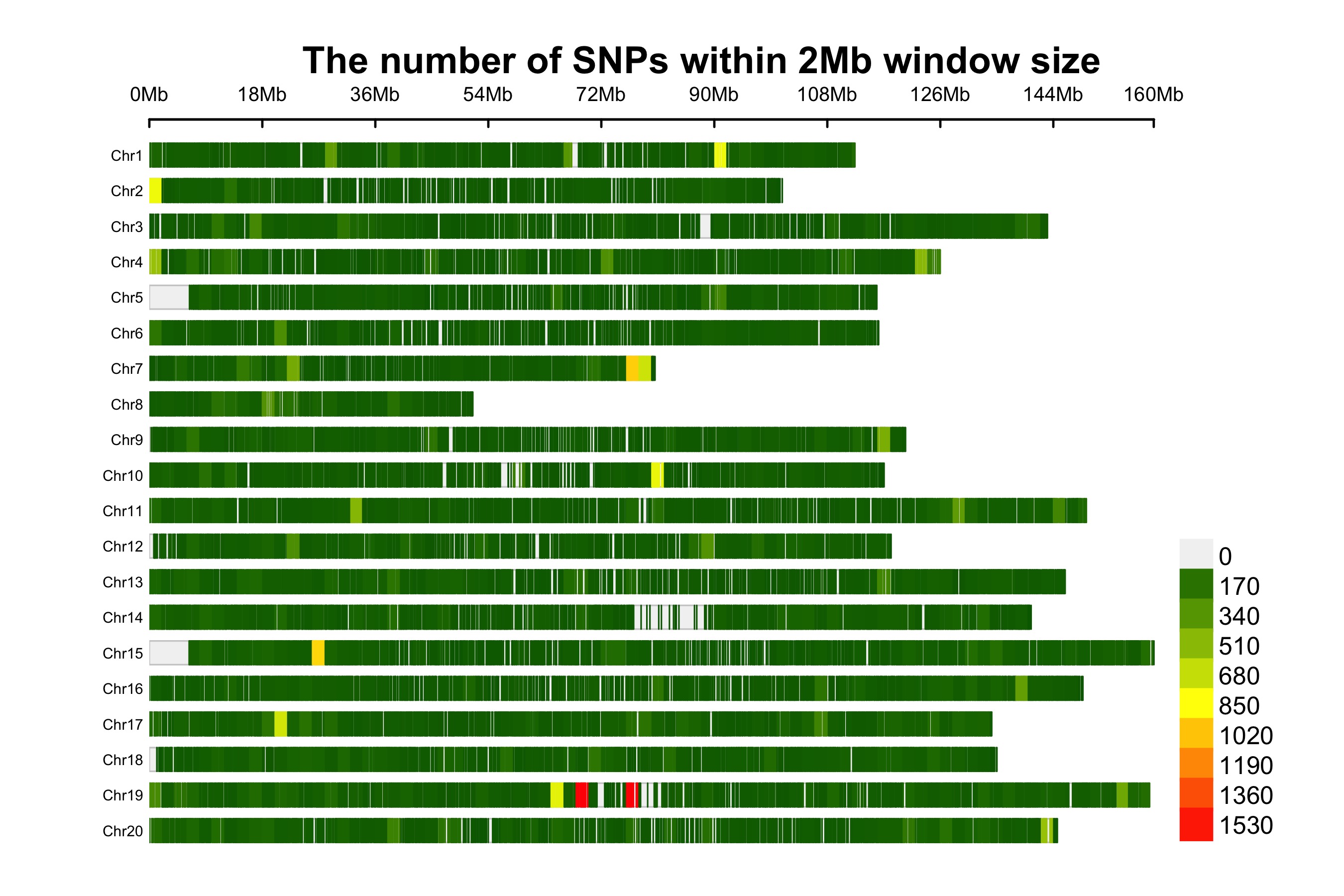

Supplement: Supplementary Figure 1 — Distribution of SNP markers in the 20 chromosomes. Horizontal axis represents physical distance along each chromosome. Vertical axis represents the 20 chromosomes of Arachis hypogaea; The color index represents the number of SNPs in 2.0 Mb window. [file Image1.jpeg]

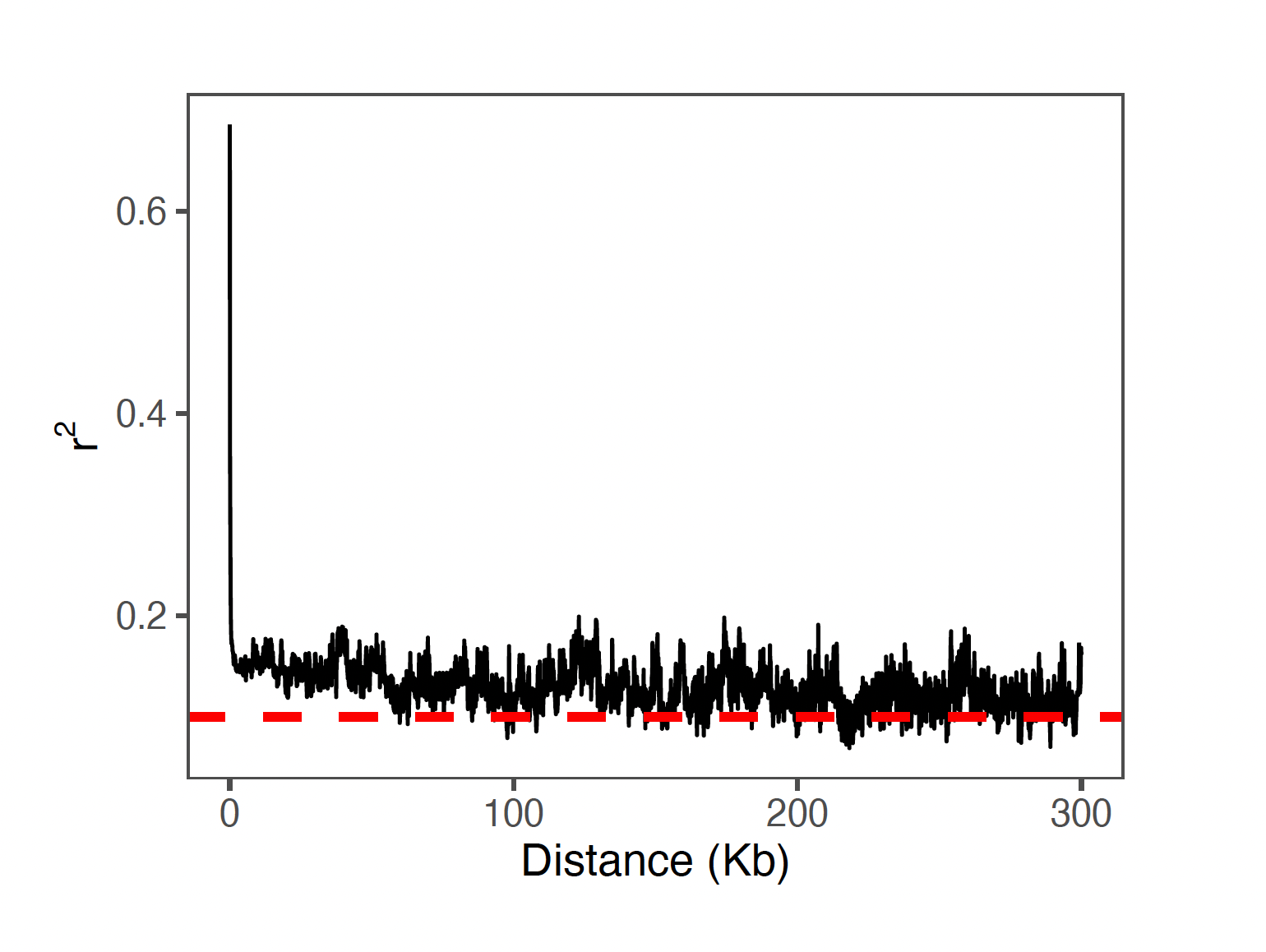

Supplement: Supplementary Figure 2 — Linkage disequilibrium (LD) decay over distance. [file Image2.tiff]

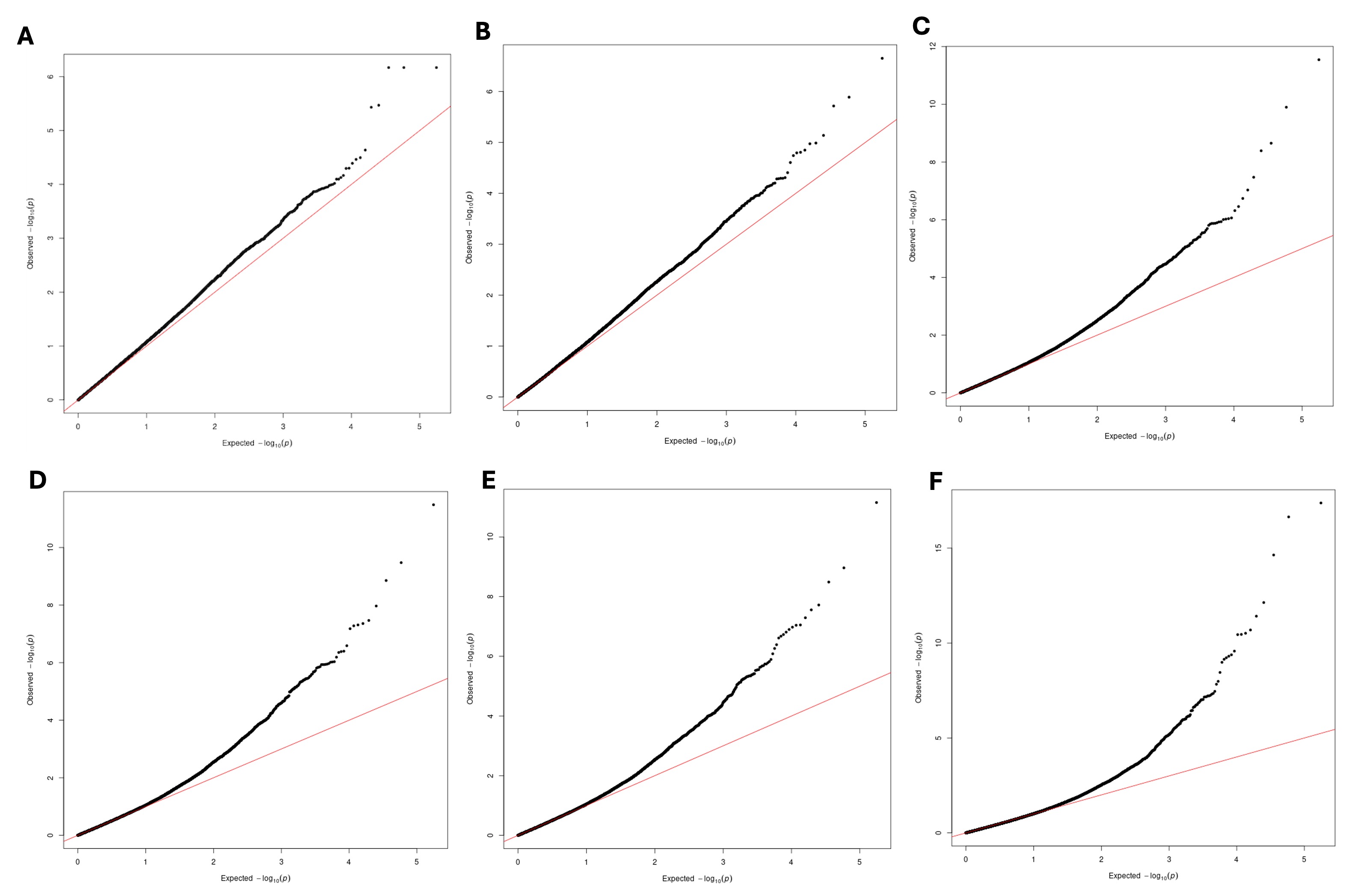

Supplement: Supplementary Figure 3 — Q–Q plot of observed vs. expected log10P values. (A) ELS. (B) LLS. (C) 7-day germination. (D) 14-day germination (E) 21-day germination (F) seed dormancy. [file Image3.jpeg]
