# Supplementary material for: Whole genome-wide association study reveals genetic insights into leaf spot disease resistances and seed germination/dormancy in peanut
Source: Front Plant Sci. 2026 Jun 10;17:1838203. doi: 10.3389/fpls.2026.1838203 (PMC13290452; doi:10.3389/fpls.2026.1838203)
Supplement: Supplementary file 4 [file DataSheet1.pdf]

Table 1: ANOVA results for ELS

Source	Sum of Squares	df	Mean Square	F	p-value
Group	56.24	2	28.12	56.96	5.30×10^{-22}
Residual	164.40	333	0.49	–	–

Table 2: Tukey's HSD post hoc test for group comparisons of ELS

Comparison	Mean Difference	95% CI (Lower, Upper)	Adjusted p-value	Significant
Group1 vs Group2	-0.398	(-0.648, -0.147)	0.0006	Yes
Group1 vs Group3	-0.926	(-1.132, -0.720)	< 0.0001	Yes
Group2 vs Group3	-0.528	(-0.765, -0.292)	< 0.0001	Yes

Table 3: ANOVA results for LLS

Source	Sum of Squares	df	Mean Square	F	p-value
Group	268.70	2	134.35	80.20	1.66×10^{-31}
Residual	980.02	585	1.68	–	–

Table 4: Tukey's HSD post hoc test for group comparisons of LLS

Comparison	Mean Difference	95% CI (Lower, Upper)	Adjusted p-value	Significant
Group1 vs Group2	-0.610	(-0.957, -0.262)	0.0001	Yes
Group1 vs Group3	-1.523	(-1.810, -1.237)	< 0.0001	Yes
Group2 vs Group3	-0.914	(-1.242, -0.585)	< 0.0001	Yes

Table 5: ANOVA results for 7th Day Germination

Source	Sum of Squares	df	Mean Square	F	p-value
Group	139900.86	2	69950.43	92.83	1.22×10^{-27}
Residual	122820.92	163	753.26	–	–

Table 6: Tukey's HSD post hoc test for group comparisons of 7th Day Germination

Comparison	Mean Difference	95% CI (Lower, Upper)	Adjusted p-value	Significant
Group1 vs Group2	-8.83	(-22.95, 5.28)	0.3031	No
Group1 vs Group3	-61.26	(-72.70, -49.83)	< 0.0001	Yes
Group2 vs Group3	-52.43	(-65.82, -39.03)	< 0.0001	Yes

Table 7: ANOVA results for 14th Day Germination

Source	Sum of Squares	df	Mean Square	F	p-value
Group	132907.42	2	66453.71	79.93	6.42×10^{-25}
Residual	135510.48	163	831.29	–	–

Table 8: Tukey's HSD post hoc test for group comparisons of 14th Day Germination

Comparison	Mean Difference	95% CI (Lower, Upper)	Adjusted p-value	Significant
Group1 vs Group2	-11.09	(-25.91, 3.74)	0.1834	No
Group1 vs Group3	-60.42	(-72.43, -48.41)	< 0.0001	Yes
Group2 vs Group3	-49.34	(-63.41, -35.27)	< 0.0001	Yes

Table 9: ANOVA results for 21st Day Germination

Source	Sum of Squares	df	Mean Square	F	p-value
Group	114826.67	2	57413.34	76.62	3.48×10^{-24}
Residual	122139.52	163	749.32	–	–

Table 10: Tukey's HSD post hoc test for group comparisons of 21st Day Germination

Comparison	Mean Difference	95% CI (Lower, Upper)	Adjusted p-value	Significant
Group1 vs Group2	-9.38	(-23.46, 4.70)	0.259	No
Group1 vs Group3	-55.90	(-67.31, -44.50)	< 0.0001	Yes
Group2 vs Group3	-46.52	(-59.88, -33.17)	< 0.0001	Yes

Table 11: ANOVA results for Seed Dormancy

Source	Sum of Squares	df	Mean Square	F	p-value
Group	107717.48	2	53858.74	68.51	2.54×10^{-22}
Residual	128135.10	163	786.35	–	–

Table 12: Tukey's HSD post hoc test for group comparisons of Seed Dormancy

Comparison	Mean Difference	95% CI (Lower, Upper)	Adjusted p-value	Significant
Group1 vs Group2	10.63	(-3.79, 25.05)	0.1922	No
Group1 vs Group3	54.57	(42.90, 66.25)	< 0.0001	Yes
Group2 vs Group3	43.95	(30.26, 57.63)	< 0.0001	Yes
